# Delayed Respiratory Insufficiency and Extramuscular Abnormalities in Selenoprotein N-Related Myopathies

**DOI:** 10.3389/fneur.2021.766942

**Published:** 2021-11-19

**Authors:** Shu Zhang, Lin Lei, Zhirong Fan, Shengyao Su, Jianying Duo, Qinrong Luan, Yan Lu, Li Di, Min Wang, Yuwei Da

**Affiliations:** Department of Neurology, Xuanwu Hospital, Capital Medical University, Beijing, China

**Keywords:** SELENON, Selenoprotein N, delayed respiratory insufficiency, extramuscular abnormalities, mutation

## Abstract

**Background:** Selenoprotein N-related myopathies (SEPN1-RMs) are a subset of congenital myopathies caused by mutations of Selenoprotein N gene (*SELENON* or *SEPN1*). Clinical phenotype is considered as highly consistent and little attention has been given to the extramuscular abnormalities.

**Methods:** We reported clinical, histopathological, and genetic features of four Chinese patients with SEPN1-RM and performed literature review on delayed respiratory insufficiency and extramuscular involvement.

**Results:** A total of four patients exhibited both the typical and atypical clinical features of SEPN1-RM. The classical manifestations included axial and limb girdle weakness, spinal rigidity, scoliosis, respiratory insufficiency, and multiminicore morphological lesions. However, high interindividual variability was noticed on disease severity, especially the onset of respiratory involvement. Two adult patients postponed respiratory insufficiency to the third decade of life, while two juvenile patients manifested early hypoventilation with puberty exacerbation. As atypical features, extramuscular involvement of weight gain, subcutaneous adipose tissue accumulation, intellectual disability, and mild cardiac changes were observed. Molecular findings revealed three novel mutations of *SELENON* such as c.1286_1288 del CCT, c.1078_1086dupGGCTACATA, and c.785 G>C. Ten cases with delayed respiratory insufficiency were identified from previous publications. A total of 18 studies described extramuscular abnormalities including joint contractures, alterations of body mass index (BMI), mild cardiac changes, and insulin resistance. Intellectual impairment was extremely rare.

**Conclusion:** SEPN1-RM should be considered as a differential diagnosis in adult patients with delayed respiratory involvement. Extramuscular involvement such as body composition alterations deserves more clinical attention. The novel mutations of *SELENON* widened the genetic spectrum of patients with SEPN1-RM.

## Introduction

Selenoprotein N-related myopathy (SEPN1-RM) is a subset of autosomal recessive congenital myopathies due to mutations of Selenoprotein N gene (*SELENON* or *SEPN1*) (1). Despite of muscle morphological differences, clinical phenotype was highly homogeneous, marked by congenital generalized hypotonia, axial and limb girdle weakness, spinal rigidity, scoliosis, and respiratory insufficiency disproportionate to limb weakness. Patients with SEPN1-RM usually develop respiratory failure in childhood or early adolescence, necessitating non-invasive ventilation in the second decade of life (2).

In spite of consistent manifestations, clinical severity varies between individuals. Aged patients with delayed respiratory involvement and juvenile cases with reduced lifespan sometimes occurred (3–5), providing a broad clinical spectrum. Moreover, extramuscular abnormalities, such as joint contractures, paradoxical insulin resistance, and body mass alteration, have also been observed in some patients (4, 6, 7). Lower body mass index (BMI) with fat tissue loss was often reported in patients around puberty, leading to a cachexia phenotype (8, 9). Mild cardiac changes or heart failure has been described in anecdotical cases (10). All these clues indicate a metabolic dysfunction and multisystem involvement of *SELENON* deficiency, which deserved more attention in clinical practice.

In this study, we reported four newly diagnosed Chinese patients with delayed respiratory involvement and atypical extramuscular features. Our cases and a comprehensive literature review supported SEPN1-RM to be a more heterogeneous metabolic myopathy.

## Subjects and Methods

### Patients and Clinical Evaluation

Four consecutive patients of SEPN1-RM were collected from April 2019 to May 2021 from the Xuanwu Hospital. Clinical evaluations and neurological examinations were performed by the two senior neurologists. This study was approved by the Ethics Committee of Xuanwu Hospital, Capital Medical University. Written informed consents were obtained from all the participants. Detailed medical history, routine laboratory tests, electromyogram (EMG), and MRI of the muscle were collected.

### Pathological Examination

Muscle biopsies from the biceps brachii or quadriceps femoris were obtained from four cases. Frozen sections of the mass specimen were processed according to the standard protocols and stained with hematoxylin-eosin staining (HE), Gomori Trichrome, nicotinamide adenine diphosphate hydride (NADH), succinate dehydrogenase (SDH), cytochrome oxidase (COX), periodic acid-Schiff (PAS), and ATPase for light microscopic investigations (2). Further immunohistochemical stainings with dystrophin R, C, N and desmin were processed.

### Genetic Analysis

Peripheral blood samples were collected from the patients and other family members. Genomic DNA was extracted from ethylenediamine tetraacetic acid (EDTA) blood. Mutation analysis was performed by whole-exome sequencing and confirmed by Sanger sequencing (11). Compound heterozygous mutations of *SELENON* gene were identified in all the patients and confirmed by familial segregation analysis. Variants were reported by using the complete *SEPN1/SELENON* transcript (NM_020451.2). Novel mutations were evaluated by the variant-classification guidelines of the American College of Medical Genetics and Genomics (ACMG) guidelines (12). The Genome Aggregation (http://gnomad.broadinstitute.org/) and the Clinvar (http://www.ncbi.nlm.nih.gov/clinvar/) databases were interrogated to identify the previously reported mutations and to determine variant frequency in the population. Pathogenicity of the variants was determined based on population frequency and *in silico* prediction programs (Protein Variation Effect Analyzer (PROVEAN), http://provean.jcvi.org/index.php; Polyphen-2c, http://genetics.bwh.harvard.edu/pph2/; Mutation Taster, http://www.mutationtaster.org; 1,000 Genomes, http://www.1000genomes.org; ExAC Browser, http://exac.broadinstitute.org; The Human Gene Mutation Database (HGMD), http://www.biobase-international.com/product/ hgmd; and SpliceAI, https://github.com/Illumina/SpliceAI).

### Literature Review

For a better delineation of heterogeneous phenotype and extramuscular abnormalities in patients with SEPN1-RM, literature review was performed in PubMed to find related articles from first available to August 20, 2021. Search terms included “*SELENON*” or “*SEPN1*” or “SEPN1-RM” or “Selenoprotein N-related myopathy” in all the fields. The full list of reported cases and corresponding variants was listed in Supplementary Table 1. Only cases described or mentioned delayed respiratory dysfunction or extramuscular abnormalities were abstracted in literature review part of this study. For each case related, we documented the basic information, clinical descriptions, and genetic data, if information was available. We excluded: (1) publications without English abstract, (2) reviews or fundamental studies with no clinical information, (3) clinical studies provide insufficient data, and (4) cases without genetic confirmed diagnosis. If one patient was reported in different studies, only the study providing more sufficient information was included. Only data that were explicitly stated in studies were abstracted.

## Results

### Clinical Features

A total of four male patients included in this study were aged 14–36 years including two juvenile cases (patients one and four) and two adult cases (patients two and three). Each of them was born from non-consanguineous patients with unremarkable family history. Their detailed clinical data are listed in Table 1. Age of onset ranged from birth to the first decade with initial symptom to be abnormal gait in patient one, delayed motor milestones in patient four, and poor sports performance in patients two and three. Symptoms of early myopathy were underrecognized, especially in patients two and three who led a normal life until the 3rd decade. All the patients presented with mild fatigue, axial and limb girdle weakness, scoliosis, rigid spine, and respiratory insufficiency. Clinical investigations disclosed reduced tendon reflexes, positive Gowers' sign, and waddling gait. As for auxiliary examination, normal or mild elevated serum creatine kinase (CK) levels (166–634 IU/L), reduced Forced Vital Capacity (FVC) (21.3–60%), and myopathic alterations in EMG were revealed. At 6 months follow-up, except for patient one who was still free of Non-invasive ventilation (NIV), other patients were supported with intermittent NIV and achieved improvement in daily activities.

**Table 1:** Detailed clinical features of four Chinese patients with SEPN1-RM.

	**Patient 1**	**Patient 2**	**Patient 3**	**Patient 4**
Gender	M	M	M	M
Age at examination	14y	36y	29y	14y
Age at first noticed signs	5y	6y	6y	Infancy
BMI (kg/m^2^)	23.2	25.3	25.4	19.2
Initial symptoms	Abnormal gait, frequent falls	Poor sports performance	Poor sports performance	Delayed motor milestones
Axial+proximal weakness	+	+	+	+
Scoliosis	+	+	- Mild	+
Rigid spine	+	+	+	+
Progression of weakness	Stable, aggravated around puberty	Stable	Stable	Stable, aggravated around puberty
Gowers' sign	+	+	+	+
Respiratory insufficiency	13y	35y	28y	13y
FVC (at age)	50% (14y)	27% (36y)	30% (28y)	21.3% (13y)
Age of NIV initiation	Not yet	36y	29y	14y
Serum CK (IU/L)	475	634	166	243
Thoracic deformities	-	-	-	+
Cardiac abnormalities	-	-	Cor pulmonale, II atrioventricular block	-
Intellectual disability	+	-	-	-
Thigh MRI	Sartorius seriously involved	Diffused adipose infiltration	Adipose infiltration	ND
EMG	Myopathic injuries	Myopathic injuries	Myopathic injuries	Myopathic injuries
SELENON Allele1	c.1574 T>G	c.1384 T>C	c.943 G>A	c.1406 G>A
SELENON Allele2	c.1286_1288 delCCT	c.1078_1086 dupGGCTACATA	c.785 G>C	c.802 C>T

*SEPN1-RM, Selenoprotein N-related myopathy; M, male; y, years; ND, not done; +, positive; -, negative*.

**Figure 1:**
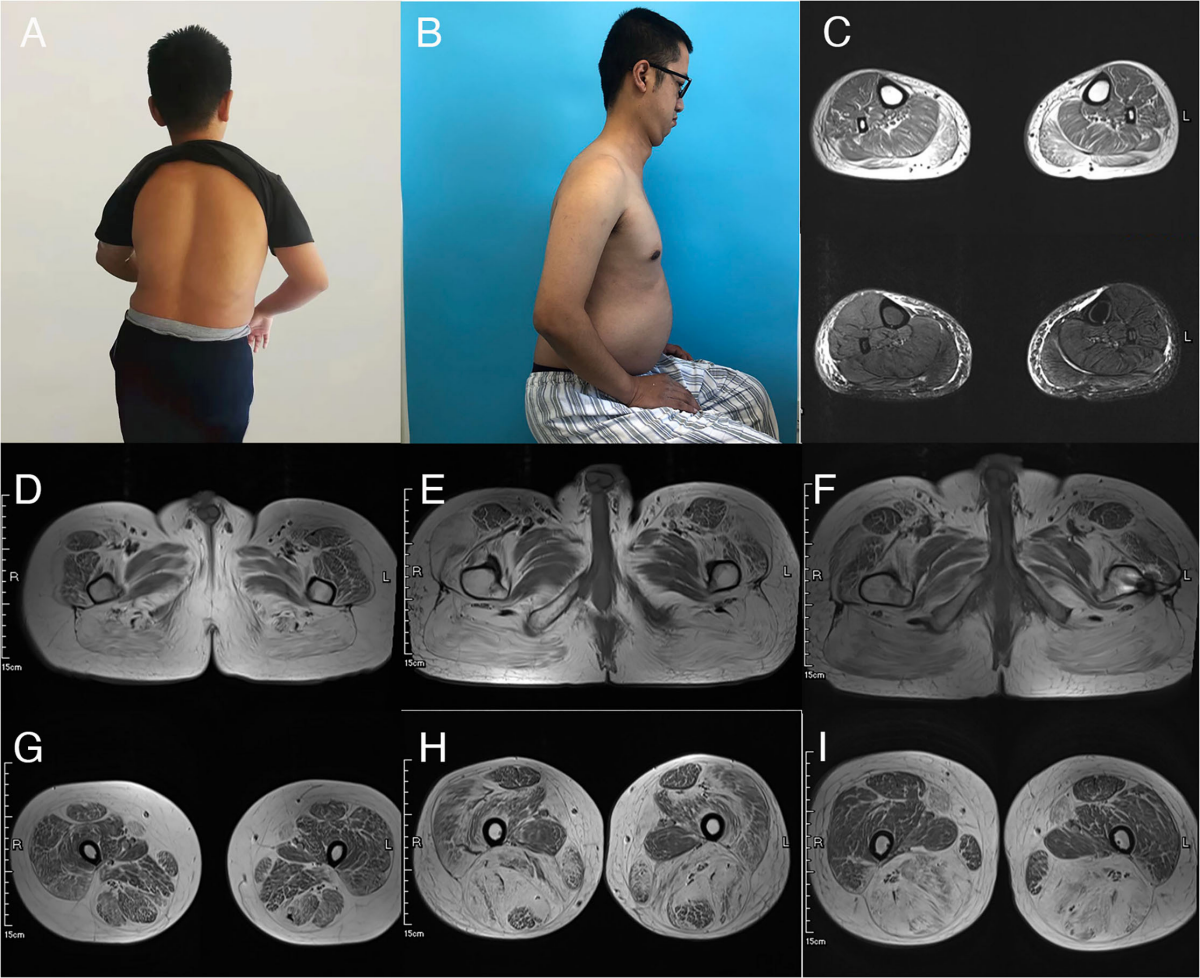
MRI findings of the thigh and photographs of patients. Scoliosis in patient one and rigid spine in patient two were showed in **(A,B)**. **(C)** Showed adipose infiltration of bilateral gastrocnemius in patient three in T2-weighted image (T2WI) (upper) and fat-saturated T2-weighted sequence (lower). Abnormal signals in bilateral gluteus maximus and subcutaneous adipose accumulation were revealed in patient one **(D)**, patient two **(E)**, and patient three **(F)**. MRI of the thigh muscle of patient 1 showed overall thigh muscle mass atrophy and diffused abnormal signal. Bilateral sartorius was the most severely involved and almost replaced by adipose tissue **(G)**. Patient two showed massive involvement of thigh muscles **(H)** with overall fat infiltration, except the rectus femoris and adductor longus. The quadriceps involvement was severe and asymmetrical, left more than right. Thigh muscles from patient three showed severe fat infiltration particularly in the posterior compartment and bilateral sartorius. Other muscles were relatively preserved **(I)**.

Clinical severity and disease progression vary between individuals. The two adolescent patients exhibited early myopathy and puberty aggravation. Patient one presented abnormal gait and suffered frequent falls at 5 years old. Scoliosis developed at the age of 12 years (Figure 1A) and slight night apnea appeared at the age of 14 years. Spinal rigidity was observed in cervical segment with limited mobility in head rotation. Patient four developed nocturnal hypoventilation, episodes of pulmonary infections, and hypoxemic and hypercapnic respiratory failure at the age of 14 years. During hospitalization, he experienced hypercapnic coma and relieved after assisted ventilation. Mild scoliosis and spinal rigidity were found. The two adult patients showed a chronic disease progression. Patient two felt normal until the age of 34 years when noticing difficulty in raising up after bending over. Later, he developed spine rigidity (Figure 1B), proximal lower limbs weakness, and progressively nocturnal dyspnea. He was diagnosed and initiated NIV at the age of 37 years. Patient three was a 29-year-old male with lower limbs weakness from the age of 22 years. He exhibited progressively nocturnal dyspnea and started NIV at the age of 29 years. No prominent scoliosis and spine rigidity were found.

Extramuscular abnormalities were observed and raised challenge for diagnosis in patients one to three. Typical features of low body weight and overall amyotrophy in SEPN1-RM were not observed in our patients. On the contrary, weight gain and fat tissue accumulation were revealed in three patients. Patient one had good appetite and gained much weight during puberty. He was a short stature with a BMI of 23.15 kg/m^2^ (height 152 cm and weight 53.5 kg) (13). Subcutaneous adipose tissue was accumulated in his chest. Patients two and three both experienced a rapid weight gain before symptoms worsened with increased dietary intake and reduced daily activities (patient two: from 53 to 73 kg at the age of 34 years; patient 3: from 50 to 65 kg at the age of 21 years). They both showed prosperously swollen abdomen (BMI of 25.3–25.4 kg/m^2^, overweight). Auxiliary examination ruled out ascites, cardiac dysfunction, thyroid problems, and diabetes mellitus. The second extramuscular symptom was mental disability. Upon entering primary school, patient one showed bad learning performances. Simple addition and subtraction within 10 were incapable. Recognition impairment was confirmed by low scores of the Wechsler intelligence question (IQ) test (IQ = 45). MRI of the brain and metabolic screening revealed no significant findings. In addition, mild cardiac abnormalities were revealed in patient three. Cardiac ultrasonography showed slightly enlarged right atrium (42 mm) and severe pulmonary arterial hypertension (72 mm Hg). Holter monitoring revealed sinus bradycardia, nodal tachycardia, and intermittent second-degree atrioventricular block (Supplementary Figure 1).

### Magnetic Resonance Imaging and Pathological Findings

**Figure 2:**
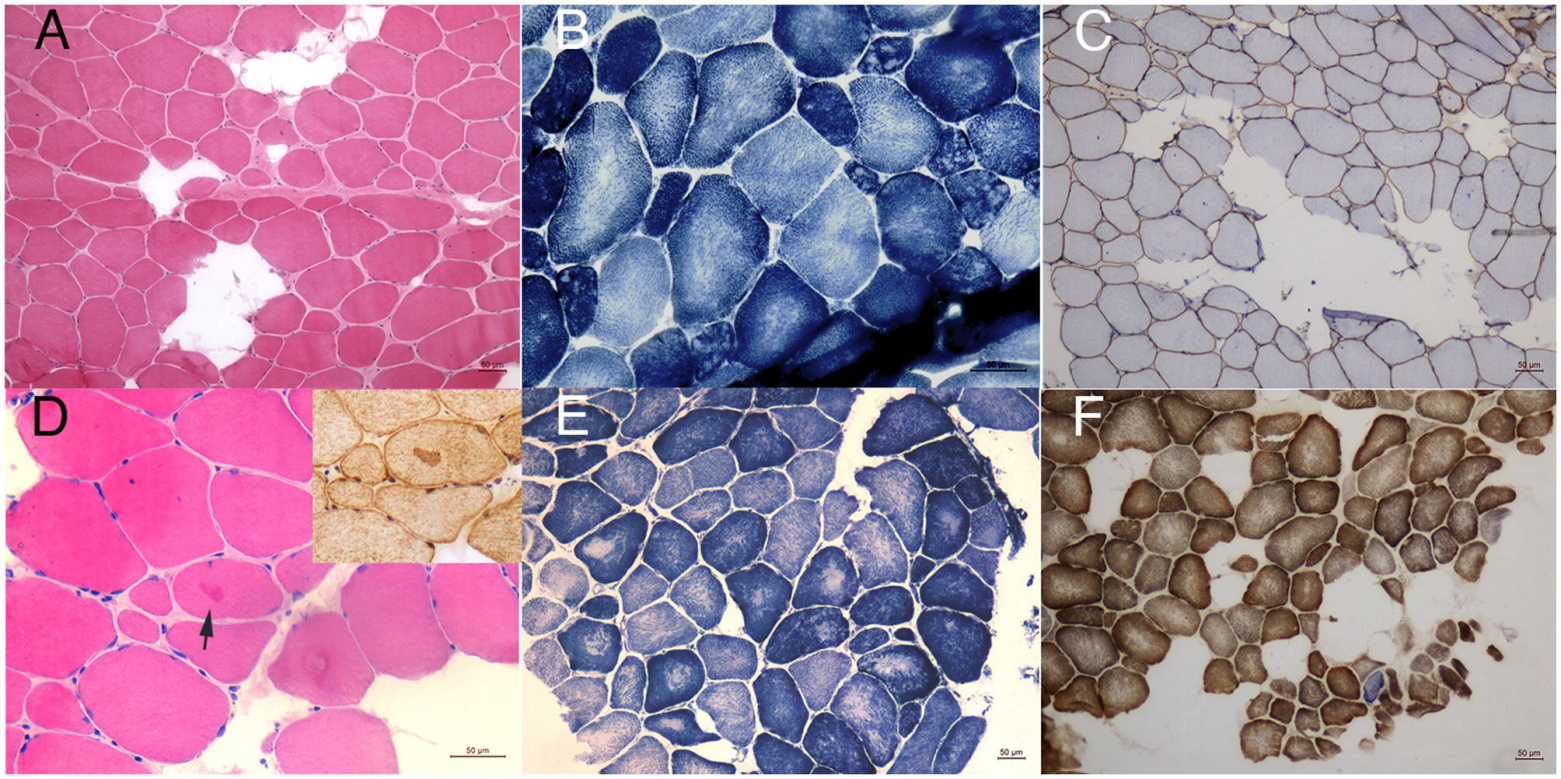
Pathological findings. HE staining of patient one **(A)** showed mild dystrophy changes with many fibers replaced by adipose tissues. NADH staining **(B)** revealed focal decrease of oxidative enzyme activities in atrophy fibers compatible of multiminicore lesions. Immunohistochemical staining of dystrophin N was normal **(C)**. In biopsy of patient four, eosinophilic inclusions could be occasionally seen in HE staining, which was immunoacitve for desmin **(D)**. His core lesions in NADH staining were relatively large compared to other patients **(E)**. SDH/COX staining of patient one **(F)** showed occasionally blue fiber (magnification 10X).

Magnetic resonance imaging of the muscle was performed in patients one to three and revealed similar involvement pattern with various severities (Figures 1C–I). Muscle mass was overall reduced and subcutaneous adipose was accumulated. Bilateral gluteus maximus (Figures 1D–F) and sartorius (Figures 1H,I) were the most commonly affected, while adductor longus and rectus femoris were often preserved even in severe lesions. The posterior compartment tends to be more seriously involved. The MRI profile was compatible of previous findings and showed no correlation with clinical severity (14–16). All the patients underwent muscle biopsy and pathological examination. The most prominent feature was the multiminicore lesions in NADH and SDH staining, which was observed in all the patients. Core changes were mostly distributed in atrophy fibers with different sizes. Other unspecific morphological changes included fiber size variations, mild increased connective tissues, type 1 fiber hypotrophy, eosinophilic inclusions immunoactive for desmin, and occasional blue fibers. Immunohistochemical staining of dystrophin was normal in all the patients. The pathological features are illustrated in Figures 2A–F.

### Genetic Findings

In all patients, compound heterozygous mutations of *SELENON* gene were identified by whole-exome sequencing, which were inherited from their parents, respectively. Three novel mutations and their pathogenic classification are listed in Table 2. All of them were absent from the control database. Patient one carried a reported pathogenic mutation c.1574 T>G (17) and a novel in-frame mutation c.1286_1288 del CCT. The latter induced a deletion of serine in 429 residue in a non-repeat region, expecting to produce a protein missing a single amino acid (p.S429del). The deleted serine was located upstream the UGA codon and not conserved itself. However, it is included within a block of strictly conserved residues, likely inducing a direct effect on Selenoprotein N structure and activity. Patient two identified a previously reported critical selenocysteine mutation c.1384T>C (p.U462R) (18) and a novel duplication c.1078_1086dupGGCTACATA (p.I362_P363insGYI). His sister was a healthy carrier of the novel mutation. It caused an in-frame insertion of the glycine, tyrosine, and isoleucine, likely producing a longer protein of 593 amino acids. It was predicted to have deleterious effects. Patient three carried heterozygous mutations of c.943 G>A and c.785 G>C. The first has been reported pathogenic (19). The latter was a novel missense mutation inducing a substitution of the highly evolutionary conserved arginine to a proline at codon 262 (p.R262P). This amino acid change constitutes a major physicochemical modification and was predicted to be disruptive on protein structure and function. Patient four identified c.1406G>A(p.R469Q) and c.802 C>T(p.R268C) of *SELENON* gene, both were reported in SEPN1-RM (5, 20). His healthy sister has not been tested. All the three novel mutations had probably no impact on splicing based on SpliceAI prediction (SELENON chr1: 26139181-26139184 TCCT>T SpliceAI = T|SELENON|0.00|0.00|0.00|0.00|39|-3|-16|-4, chr1: 26138020 dup A AGGCTACATA SpliceAI = AGGCTACATA|SEPN1|0.00|0.00|0.00|0.00|0|7|33|6, chr1: 26135554 G>C SpliceAI = C|SEPN1|0.05|0.01|0.00|0.00 |14|-37|-38|-16).

**Table 2:** Novel mutations detected in four patients with SEPN1-RM and their pathogenesis classification.

**Genomic alteration**	**chr1:26139181-26139184**	**chr1:26138011**	**chr1-26135554**
Nucleotide alteration	c.1286_1288 delCCT	c.1078_1086 dupGGCTACATA	c.785 G>C
Amino acid alteration	p.S429del	p.I362_P363insGYI	p.R262P
Exon	10	8	6
Exac	-	-	-
1000 Genomes	-	-	-
PROVEAN	Deleterious (Score: −7.24)	Deleterious (Score: −8.00)	Deleterious (Score: −4.39)
Polyphen2	-	-	Probably damaging
Mutation taster	Disease causing	Disease causing	Disease causing
Conervation	-	-	Highly conserved
Clinvar	No	No	No
HGMD	No	No	No
ACMG interpretaiton	VUS	Likely pathogenic	VUS

### Literature Review

In the literature review, we found 10 cases of SEPN1-RM presenting delayed respiratory insufficiency (Table 3) (2–4, 10, 21–23). These patients were globally distributed and ranged from 26 to 71 years old. Nocturnal dyspnea and NIV assistance were postponed to the third to eighth decades of life, sometimes after predisposing events such as stroke, acute respiratory failure, and pregnancy. All these patients presented relatively mild symptoms of an infantile myopathy including muscle weakness, spinal rigidity, scoliosis, and contractures. No correlation was found between the mutation type and clinical severity.

**Table 3:** Delayed respiratory insufficiency in patients with SEPN1-RM.

**ID**	**Origin**	**Year**	**Gen**	**Age**	**Early symptoms**	**Predisposing factor**	**Respiratory involvement**	**Mutations of SELENON**	**References**
1	China	2021	M	36	Wea, Rig	Weight gain	36ys	c.1384T>C + c.1078_1086dupGGCTACATA	This study
2		2021	M	29	Wea	Weight gain	28ys	c.943 G>A + c.785 G>C	This study
3	Japan	2021	F	71	Wea	None	71ys	homozygous c.227T>C	(3)
4	INT	2020	F	31	Wea	Stroke	31ys	NK	(4)
5	USA	2019	NA	34	Wea, Ske	NK	32ys	NK	(21)
6	UK	2011	M	59	Rig	Acute RF	33ys	c.802C>T + c.1358G>C	(22)
7			M	40	Wea	Acute RF	33ys	c.943G>A + c.1315C>T	(22)
8	Italy	2011	F	32	Wea, Hyp	None	32ys	c.19_73del92 + c.1176delA	(2)
9	Syrian	2006	F	45	Wea, Rig, Sco	Pregnancy	34ys	Homozygous g.17195T>C	(23)
10	UK	2005	M	26	Wea, Rig, Sco, Con	None	26ys	Homozygous c.943G>A	(10)

*Gen, gender; ys, years; Weak, proximal and axial weakness; Rig, spine rigidity; Sco, scoliosis; Hyp, hyperlordosis; Con, contractures; Ske, skeletal abnormalities; NK, not known; RF, respiratory failure; Ref, reference; C, childhood; INT, international case series*.

There were 18 studies mentioned extramuscular abnormalities of SEPN1-RM in current literature (Table 4). Since this aspect has rarely been focused, literature review only provided a brief overview rather than quantitative analysis. Overall, joint contractures/hyperlaxity and body composition abnormalities seem to be the most common extramuscular symptoms (4). In patients with altered body composition, low BMI/body weight was frequently reported, while overweight cases or steep weight gain were quite rare (4, 22). Some patients showed short stature and altered fatty tissue distribution (6, 14). Additional metabolic changes included insulin resistance and osteoporosis (6, 7, 28). A few patients presented cardiac abnormalities of which mostly were mild or secondary to respiratory failure (4, 10, 22, 28). Three cases combined intellectual disability or developmental delay (8, 18).

**Table 4:** Clinical data of the reported SEPN1-RN in patients mentioned extramuscular abnormalities.

**ID**	**Origin**	**Year**	**Total case**	**Age (range)**	**Gen (M/F)**	**Description of extramusclar abnormalities**	**References**
1	China	2021	4	14–36	M	Body composition abnormalities (3), short stature (1), intellectual disability (1), cardiac abnormalities (2)	This study
2	International case series	2020	60	2–58	29/31	Underweight (16), steep weight gain (2), feeding difficulty (10)	(24)
3	Iran	2020	1	9	M	Intellectual disability	(8)
4	International case series	2020	132	2–58	65/67	Cardiac abnormalities (15.79%), joint contractures /hyperlaxity (64.37%), mild learning difficulties (3), body composition abnormalities (72.7% underweight, 2 overweight, 2 obese)	(4)
5	China	2020	1	16	M	Joint contractures	(11)
6	Italy	2019	8	22–52	4/4	Insulin resistance (4), low BMI (<13.1) (4)	(7)
7	Iran	2019	1	14	M	Joint contractures, cachectic with low BMI (BMI 9.18)	(25)
8	India	2018	1	7	M	Hyperlaxity and contractures	(26)
9	Italy	2016	3	4–16	2/1	Short stature (1), growth hormone deficiency (1)	(6)
10	China	2016	1	17	M	Delayed psychomotor development	(18)
11	Saudi Arabia	2015	1	10	M	Altered fatty tissue distribution	(14)
12	UK case series	2011	41	1–60	23/18	Mild cardiac abnormalities (5), body composition abnormalities (26 underweight and 3 overweight), joint contractures (26)	(22)
13	Germany case series	2008	11	1–16	3/8	Low BMI (<20) (11), joint contractures (1)	(27)
14	UK	2006	1	26	M	Right heart failure, moderate pulmonary hypertension	(10)
15	Australia	2006	8	18–32	F	Cardiac abnormalities (2), insulin resistance (5), Osteoporosis (2), low body weight (1)	(28)
16	International case series	2004	6	21–35	4/2	Joint contractures (4), secondary heart failure(2)	(29)
17	International case series	2002	6	13–22	NA	Cardiac failure (1), joint contractures/hyperlaxity (5/1), midface hypoplasia (2)	(16)
18	International case series	2002	17	6–41	9/8	Short stature (16), low body weight (16), joint contractures (half)/hyperlaxity (most)	(19)

*Gen, gender; M, male; F, female*.

*Number of each symptom was indicated as numbers in brackets*.

## Discussion

Selenoprotein N-related myopathy is usually associated with a typical signature of congenital myopathy (4, 30). However, clinical spectrum might be broader and more heterogeneous than previously considered. In this study, four Chinese patients with different clinical severities and atypical manifestations were investigated. They had either delayed respiratory insufficiency or extramuscular abnormalities, especially weight gain, which was rare in patients with *SELENON* deficiency. Three novel mutations were also identified.

Respiratory insufficiency, mostly emerged in childhood or adolescent in SEPN1-RM, was delayed in two current adult patients. Respiratory involvement from adulthood was unusual with only few cases reported as reviewed in Table 3. Clinical phenotype could be divided into three categories according to the onset of respiratory insufficiency: (i) the severe type, in which respiratory insufficiency and mechanical ventilation occurred during infancy or early childhood (within 10 years), usually with a poor prognosis (4, 5, 22, 31). As the more malignant end of the spectrum, disease could be severe enough to cause systematic functional decline and reduced lifespan; (ii) the classical type, in which respiratory insufficiency developed in adolescence (during the second decade of life). This is a classical type because most cases manifested full phenotype around puberty and 81.9% initiated assisted ventilation at a mean age of 14.14 years (4). Patients one and four reported here belonged to this type; (iii) the mild type, characterized by delayed onset of respiratory insufficiency, often postponed to the adulthood, with a stable or chronically progressive disease course (2–4, 10, 21–23). At the more benign end of the clinical spectrum, there was clinical presentation of patients two and three, of whom respiratory insufficiency emerged particularly late after a prolonged compensation. No genotype–phenotype correlation was found. The high interindividual variability indicated the involvement of other genetic and environmental factors in modifying disease phenotype.

The heterogeneity also manifests in extramuscular abnormalities, which were often overlooked. Based on literature review, joint contratures/hyperlaxity and altered body composition were probably the most frequent extraskeletal features. However, none of our patients was disclosed of any joint contractures or hyperlaxity. Low body weight has been considered as a core feature in patients with SEPN1-RM (4). Conversely, patients in this study showed a different profile of weight gain and fat accumulation. Retrospectively, overweight cases and adipose accumulation have been sporadically reported by the international cases series and revealed through whole-body MRI profile (4, 14, 22). We supposed weight gain in these patients that might be ascribed to reduced activity levels, increased diary intake, and a potential metabolic disturbance underlying SEPN1 deficiency. Murine models of *SELENON* deficiency showed similar phenotype of reduced lean mass, increased fat tissue, and higher food intake (9). However, loss of subcutaneous fat and extremely low BMIs were more often observed around puberty in patients with SEPN1-RM, leading to a cachexia phenotype (4, 7, 25). Consistently, adult SEPN1 RO mice showed age-related body weight loss, despite high food intake (9). These seemingly contradictory observations might be two sides of the same coin. It is possible that dietary intake alteration, body composition changes, and insulin resistance were all the manifestations of reduced bioenergetics efficiency. Certain symptom might be dominant at one time and transformed to another as disease progression, resulting in overweight/fat accumulation in some patients and weight loss/cachexia in other patients.

In fact, there is evidence that *SELENON* played a role in lipid metabolism and mitochondrial biogenesis and its depletion increased susceptibility to insulin resistance and toxicity of saturated fatty acids (7, 9, 32, 33). Enriched at endoplasmic reticulum (ER)-mitochondria contact sites mitochondria-associated membranes (MAM), *SELENON* is required for ER-to-mitochondria calcium transfer and energy production (9, 34). Consistent with this, fatigue and defective exercise endurance in patients with SEPN1-RM was a typical feature of metabolic myopathy with compromised ATP production. Moreover, subsequent functional decline after weight gain in our patients was in line with the correlation between body weight and disease severity (4). It was plausible that fat accumulation and aggravated metabolic stress would increase compensatory load and accelerate functional deterioration. Unfortunately, we did not perform glucose tolerance test in our patients. So, our cases should be under careful follow-up. Further study is also needed to better assess body composition and nutritional status of patients with SEPN1-RM.

Notably, cognitive impairment in patient one was very uncommon in SEPN1-RM. To the best of our knowledge, there was only one well-described child from Iran reported moderate intellectual disability with an IQ of 48 (8). Another Swedish case mentioned intellectual development disorder without detailed information (35). Several cases showed mild learning difficulties or developmental delay with no explicit mental deficiency (4, 18). It was uncertain whether the cognitive disability in patient one was a rare phenotype of SEPN1-RM or a coincidence due to the relatively high frequency of cognitive impairment among general population. Intriguingly, Selenoprotein N deficiency was found to impact ryanodine receptors (RyRs) efficiency by altering its biochemical properties and that intracellular Ca^2+^ dysregulation in RyRs deficiency has been associated with Alzheimer's disease (AD) (36, 37). It is probable that *SELENON* deficiency and prolonged hypoxia might compromise intellectual development with other predisposing factors in the growing-up period.

We reported three novel mutations of *SELENON* such as c.1286_1288 del CCT, c.1078_1086dupGGCTA- CATA, and c.785 G>C. Although two of them are classified variants of unknown significance (VUS) according to the ACMG guidelines, all were predicted pathogenic *in silico* studies. Furthermore, the clinical phenotype, muscle involvement pattern, and multiminicore changes were all compatible of SEPN1-RN, making it currently the most probable diagnosis. SEPN1 played an important role in oxidative stress protection and redox-related calcium homeostasis (38). Mutations related to predicted functional motifs might be pathogenic including the putative reductase domain, calcium-interacting domain, and transmembrane and several glycosylation sites (1, 34). Overall, Chinese SEPN1-RM cases were only sporadically reported, seemingly extremely rare compared to the Caucasians (18, 31, 39, 40). To date, c.1384 T>C in exon 10 has only been identified in Chinese population (18, 39) and was absent from control database. Though the accurate allele frequency was unavailable, this variant might have a higher carrier rate in Chinese population. Missense mutations encoding SCUG sequence were mostly associated with moderate severity (4). In large broad case series, exon 1 harbored the highest number of variants and were frequently identified in severe phenotype (4). The ethnic variation in hypermutable region, environmental modifiers, and a bias from small sample size might contribute to the mild phenotype in patients with SEPN1-RM, which should be confirmed in larger number of patients in the future. Additional studies are needed to have a proper genotype–phenotype correlation.

In summary, the clinical phenotype of patients with SEPN1-RM was not so homogeneous as considered and interindividual variability was high. Respiratory insufficiency and NIV assistance might be postponed to adulthood. Extramuscular symptoms such as body mass alterations reflected an underlying metabolic disturbance, which deserved more attention.

## Data Availability Statement

The original contributions presented in the study are included in the article/Supplementary Material, further inquiries can be directed to the corresponding author/s.

## Ethics Statement

The studies involving human participants were reviewed and approved by the Institutional Ethics Committee of Xuan Wu Hospital, Capital Medical University. Written informed consent to participate in this study was provided by the participants' legal guardian/next of kin. Written informed consent was obtained from the individual(s) for the publication of any potentially identifiable images or data included in this article.

## Author Contributions

SZ contributed to drafting and revising the manuscript, study design, and acquisition of data. ZF, LL, and SS contributed to acquisition of data. JD and QL contributed to pathological staining. YL, LD, and MW contributed to pathological image analysis and genetic data interpretation. YD contributed with drafting and revising the manuscript, study design, and interpretation of the data. All the authors contributed to the article and approved the submitted manuscript.

## Funding

This study was supported by the National Key R&D Program of China, the Precision Medicine Project (2017YFC0907700), the National Natural Science Foundation of China (82001352, 81801255), and the Beijing Hospitals Authority Youth Program (QML20190803).

## Conflict of Interest

The authors declare that the research was conducted in the absence of any commercial or financial relationships that could be construed as a potential conflict of interest.

## Publisher's Note

All claims expressed in this article are solely those of the authors and do not necessarily represent those of their affiliated organizations, or those of the publisher, the editors and the reviewers. Any product that may be evaluated in this article, or claim that may be made by its manufacturer, is not guaranteed or endorsed by the publisher.

## Supplementary Material

Supplementary Figure 1 24h Holter monitoring electrocardiogram of patient 3 showed intermittentsecond-degreeatrioventricularblock.
