# Supplementary figures and images for: Delayed Respiratory Insufficiency and Extramuscular Abnormalities in Selenoprotein N-Related Myopathies
Source: Front Neurol. 2021 Nov 19;12:766942. doi: 10.3389/fneur.2021.766942 (PMC8639696; doi:10.3389/fneur.2021.766942)

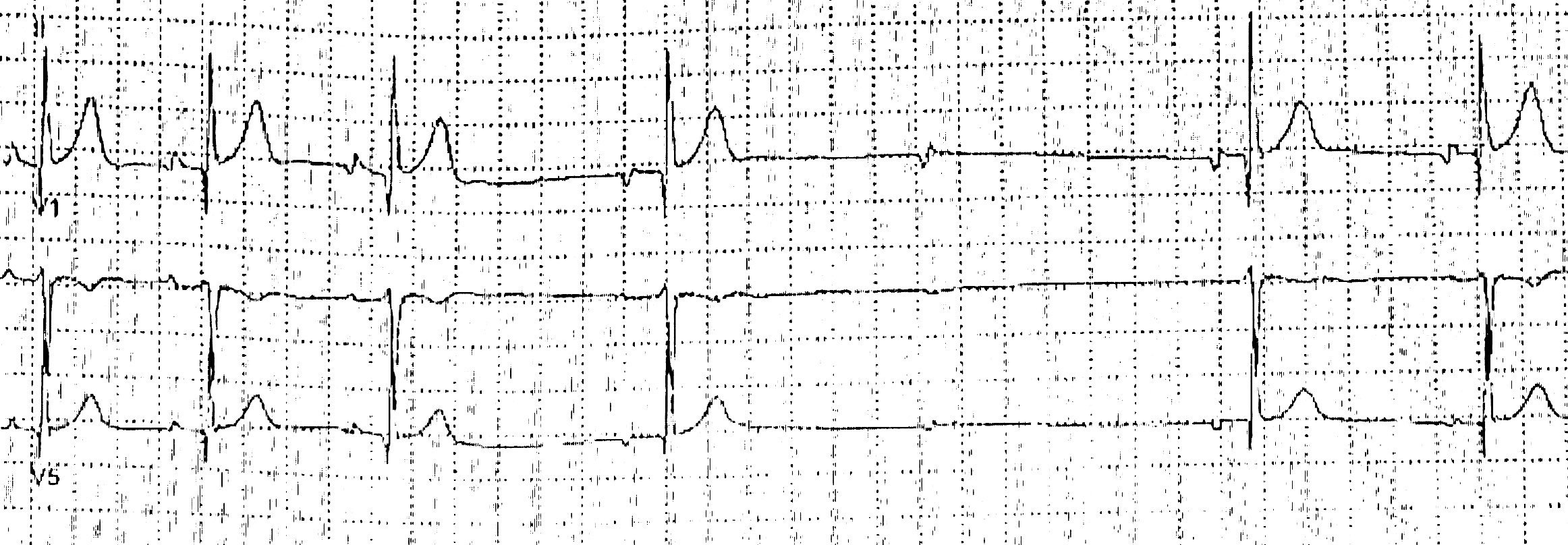

Supplement: Supplementary Figure 1 — 24h Holter monitoring electrocardiogram of patient 3 showed intermittentsecond-degreeatrioventricularblock. [file Image_1.jpeg]
